# A History of Tuberculosis

**Published:** 1883-09

**Authors:** 


**A History of Tuberculosis**, from the time of Sylvius to the present day, being in part a translation, with notes and additions, from the German of Dr. Arnold Spina; containing, also, an account of the researches and discoveries of Dr. Robert Koch and other recent investigators. By ERIC E. SATTLER, M. D. Cincinnati: Robert Clarke & Co. 1883. Price $1.25.

It is the aim of this volume to supply a history of the study of Tuberculosis, from the earliest times to the present day. The account of the investigations of Koch are peculiarly interesting, and the author has succeeded in presenting the whole subject in an exhaustive and forcible way.

